# Supplementary material for: A Global Screen for Assembly State Changes of the Mitotic Proteome by SEC-SWATH-MS
Source: Cell Syst. 2020 Feb 26;10(2):133–155.e6. doi: 10.1016/j.cels.2020.01.001 (PMC7042714; doi:10.1016/j.cels.2020.01.001)

O43505 | B4GA1_HUMAN | B4GAT1 B3GNT1 B3GNT6
Monomer MW [kDa]: 47.119 Monomer expected elution fraction: 47

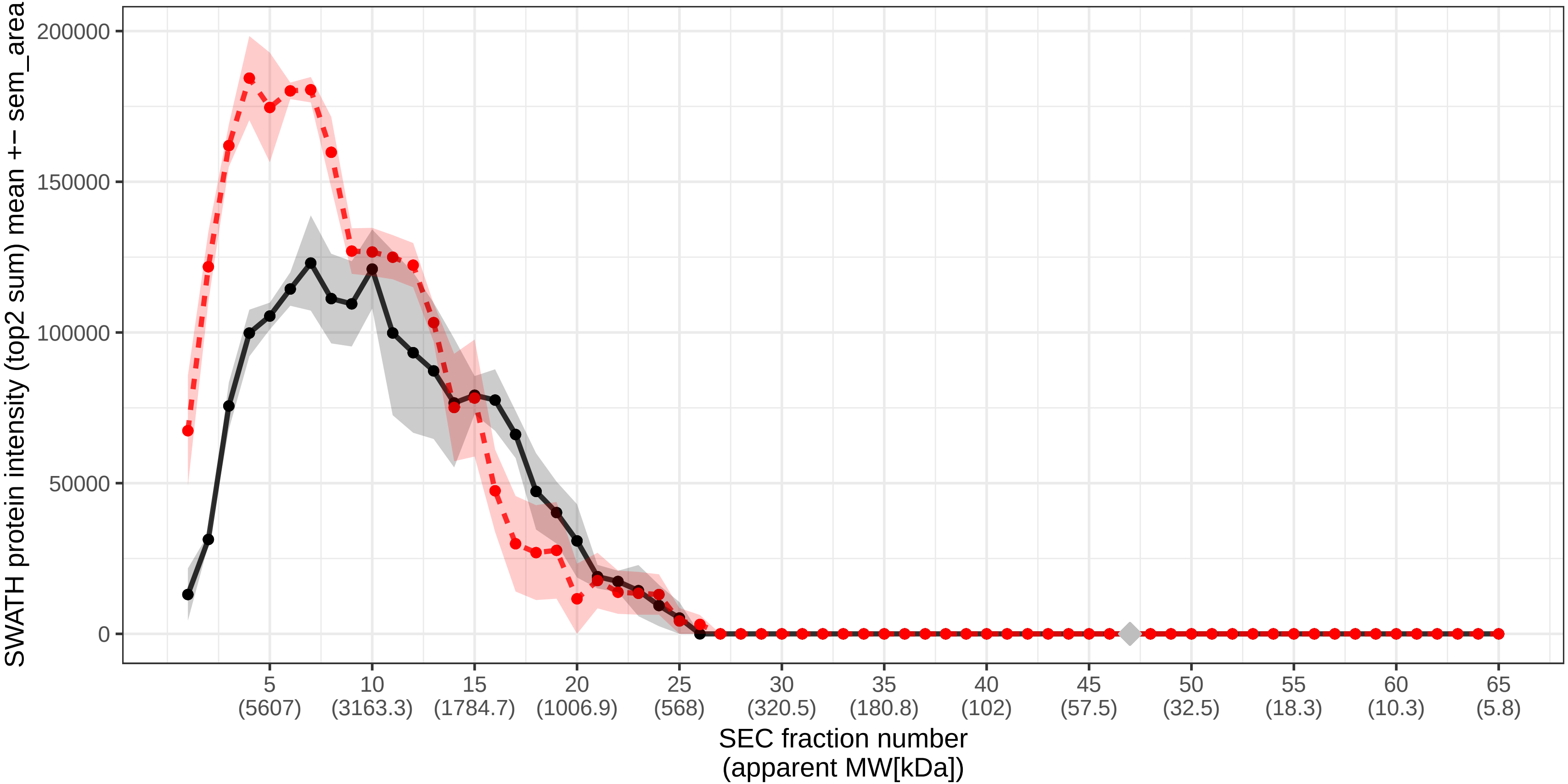

Supplement: Data S1. SEC-SWATH-MS Protein Chromatograms, Related to Figure 1 [file mmc6.zip › SECchrom_O43505_B4GA1_HUMAN_B4GAT1_B3GNT1_B3GNT6.pdf]
